# Stromal fibroblastic mutant Trp53 promotes mammary tumor development via enhanced secretion of paracrine factors

**DOI:** 10.1038/s41523-025-00876-y

**Published:** 2025-12-12

**Authors:** Bin Liu, Shunbin Xiong, Abie Williams-Villalobo, Gilda Chau, Yuan Qi, Hehua Chen, Carenza Johnson, Dhruv Chachad, Joy McDaniel, Xiaoping Su, Adel K. El-Naggar, Guillermina Lozano, Yun Zhang

**Affiliations:** 1 Department of Epigenetics and Molecular Carcinogenesis, The University of Texas MD Anderson Cancer Center, Houston, TX USA; 2 Department of Genetics, The University of Texas MD Anderson Cancer Center, Houston, TX USA; 3 Department of Pharmaceutical Sciences, Joan M. Lafleur College of Pharmacy and Health Sciences, Texas Southern University, Houston, TX USA; 4 Department of Bioinformatics & Computational Biology, The University of Texas MD Anderson Cancer Center, Houston, TX USA; 5 Department of Anatomical Pathology, The University of Texas MD Anderson Cancer Center, Houston, TX USA

**Keywords:** Cancer, Cell biology, Molecular biology, Oncology

## Abstract

Mutations in the tumor suppressor gene *TP53* have been identified in breast cancer-associated fibroblasts and are associated with poor patient prognosis. However, the functional impact of fibroblastic mutant p53 on breast cancer development remains unclear. To investigate this, we compared female mice harboring HER2-driven mammary tumors with a fibroblast-specific *Trp53* mutation (*NP*) to those with wild-type fibroblastic *Trp53* (*N*). *NP* mice exhibited significantly shorter median tumor-free survival than *N* mice. RNA sequencing of *NP* and *N* tumors and mammary glands revealed numerous differentially expressed genes (DEGs) between tumors and the corresponding glands in both genotypes. Notably, the *NP* tumors showed enrichment of several signaling pathways, including PI3K/AKT/mTOR. Additionally, fifteen DEGs encoding secreted proteins were identified between *NP* and *N* mammary glands. Among these, *SAA1* and *SAA2* were also upregulated in human breast tumors with mutant *TP53* compared to those with wild-type *TP53*. Previous studies have implicated SAA1, SAA2, and THBS4 in promoting tumor progression via the PI3K/AKT pathway. Consistently, supplementing primary HER2-positive tumor cultures with recombinant SAA1, SAA2, or THBS4 peptides enhanced tumor cell proliferation and migration. Together, these findings uncover a mechanism by which fibroblastic mutant p53 promotes mammary tumorigenesis—through upregulating secretory proteins such as SAA1, SAA2, and THBS4 in the stroma, thereby enhancing PI3K/AKT signaling and tumor progression.

## Introduction

Emerging evidence has demonstrated that malignant transformation and progression of tumor evolution require the unique and essential contribution of stroma, which is composed of the immune system, vasculature, mesenchymal supporting cells, and extracellular matrix (ECM)^1^. As the major type of mesenchymal cells and predominant constituent of the tumor stroma, fibroblasts play a profound role in tumor development by synthesizing growth and survival factors, angiogenic and immunological chemokines, and structural components of the ECM, as well as enzymes that control their turnover^2,3^. Furthermore, cancer-associated fibroblasts (CAFs) are believed to adapt and continuously co-evolve along with tumor epithelial cells to foster transformation and tumor growth^4^.

As the most frequently altered tumor suppressor in human cancer, TP53 has been extensively investigated^5^. The majority of TP53 studies have focused on its cell-autonomous functions^5^. However, emerging evidence indicates that stromal TP53 also plays an active role in tumor–stroma crosstalk (reviewed in refs. ^6,7^). For example, xenograft cancer cells form tumors with a shorter latency in *Trp53*-null mice than in wild-type (WT) mice^8^. In liver injury, p53-driven senescence in stellate cells limits fibrosis and hepatocellular carcinoma by releasing factors that steer macrophages toward tumor-suppressive activity, whereas loss of this pathway promotes fibrosis, cirrhosis, and cancer^9^. Moreover, frequent *TP53* mutation hotspots, particularly in the DNA-binding domain, such as arginine 175, 248, 249, 273, and 282 (R175, R248, R249, R273, and R282), as well as glycine 245 (G245), disrupt its canonical tumor suppressor functions and contribute to tumorigenesis and progression^10–12^. Notably, the impact of mutant TP53 also extends beyond the cell-autonomous level. For instance, fibroblasts expressing mutant Trp53 promote tumor growth more effectively than Trp53-null fibroblasts^13^, underscoring its role in shaping the tumor microenvironment. Interestingly, in another study, unlike its tumor-suppressive role in normal fibroblasts, TP53 in CAFs isolated from a lung cancer patient supports a distinct transcriptional and secretory program that promotes tumor cell migration, invasion, and growth. The tumor-promoting ability of CAFs was greatly compromised upon depletion of their endogenous WT *TP53* when co-xenografted with lung tumor cells in SCID mice^14^. These findings indicate that tumor cells may “educate” stromal p53 into a tumor-supportive state without requiring genetic mutations. In human samples, attenuation of TP53 activation has also been revealed in CAFs. Several studies described *TP53* gene mutations or loss of heterozygosity (LOH) in CAFs of various human cancers, including breast cancer, and TP53 protein expression in stromal fibroblasts is closely associated with the number of nodal metastases and disease prognosis^15–17^. However, it is important to note that the above findings, while compelling, are primarily based on xenograft models and correlative human data. Xenograft systems may not fully capture the complexity of native tumor–stroma interactions, and human studies largely reveal associations rather than direct causative relationships between stromal *TP53* alterations and tumor progression. Furthermore, little is currently known about the molecular mechanisms through which mutant TP53 in stromal fibroblasts influences tumor development.

Bridging this knowledge gap directly requires a genetic model that enables targeted in vivo induction of mutant p53 specifically in fibroblasts. We previously generated a conditional mutant *Trp53* allele, *Trp53*^*wm-R172H*^, which allows Cre recombinase-mediated conversion of WT p53-to-p53R172H mutant (corresponding to the human p53R175H, an arginine-to-histidine substitution), faithfully recapitulating how somatic *TP53* mutation occurs in clinical settings^18^. This allele has been widely used to investigate diverse somatic cancers^19–25^. In this study, using the *Trp53*^*wm-R172H*^ allele, we specifically induced mutant p53 in fibroblasts while maintaining intact WT p53 in other cellular compartments in mice. We investigated the impact of stromal fibroblast-specific p53R172H on ERBB2-driven mammary tumor development and observed that mice with this mutation developed tumors more rapidly than those with WT p53. These tumors exhibited distinct pathway enrichment, including PI3K/AKT/mTOR signaling, and elevated levels of secretory proteins such as SAA1, SAA2, and THBS4. In vitro assays confirmed that these factors enhanced the proliferation and migration of HER2-positive (HER2+) tumor cells. Our findings suggest that fibroblastic mutant p53 drives tumorigenesis by reshaping the tumor microenvironment.

## Results

### The presence of mutant p53 in stromal fibroblasts accelerates breast tumor development

To directly investigate the role of mutant p53 in stroma on breast tumor development, we generated a cohort of female mice with the following genotypes: *MMTV-neu*; *Fsp-Cre*; *Trp53*^*wm-R172H/+*^ (*NP*) and *MMTV-neu*; *Fsp-Cre or Trp53*^*wm-R172H/+*^ (*N*). Here, the *MMTV-neu* allele expresses WT ERBB2 protein in mammary epithelium, leading to ERBB2, also known as HER2+ mammary tumor in female mice^26^. *Fsp-Cre* is a transgene that expresses Cre recombinase specifically in fibroblasts^27^, and *Trp53*^*wm-R172H*^ is the conditional allele producing WT p53, which can be converted into the *Trp53*^*R172H*^ mutant allele expressing p53R172H mutant protein in the presence of Cre ^18^.

**Fig. 1:**
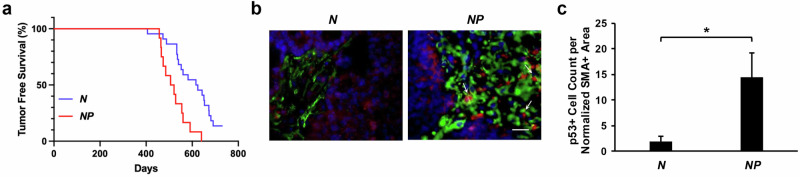
Mutant p53 in the tumor stroma accelerates breast tumor development. **a** Kaplan–Meier curves showing mammary tumor-free survival of *N* (n = 22) and *NP* (n = 12) female mice. *p* = 0.0007. **b** immunofluorescence images of mammary tumors from the *N* and *NP* female mice. Green, smooth muscle actin; Red, p53. **c** Quantification of p53-positive (p53+) cells within the normalized area of smooth muscle actin-positive (SMA+) staining in immunofluorescent images. n = 3 for each genotype. *p = 0.007.

As expected, mammary tumors developed in both *NP* and *N* females. However, *NP* mice, which harbor mutant p53 in fibroblasts, exhibited a significantly shorter median survival of 520 days compared to 620 days in *N* mice (*p* = 0.0007; Fig. 1a). Notably, the median survivals in both groups were substantially longer than previously reported, where 50% of female *MMTV-neu* carriers developed mammary tumors by 205 days^26^. This discrepancy may be at least partially attributed to differences in genetic background between our cohort (FVB/C57/BALB/c) and the originally reported *MMTV-neu* model (FVB). Stabilization of p53 was clearly observed in *NP*, but not *N*, tumor-associated fibroblasts marked by the fibroblast-specific marker α-smooth muscle actin (SMA), indicating fibroblast-specific recombination of the *p53*^*wm-R172H*^ allele in *NP* mice (Fig. 1b). Quantification further showed a significantly higher number of p53-positive cells in fibroblasts from *NP* mice compared with those from *N* mice (Fig. 1c).

All the mammary tumors are identified as adenocarcinomas pathologically. As previously reported, some tumors disseminated to the lung^26^. However, there is no significant difference in the percentage of tumors that metastasized between *NP* and *N* mice (16% vs 18%, Supplementary Fig. 1a). Consistent with previous findings, the HER2/neu positive (HER2/neu+) mammary tumors develop in multiple mammary glands simultaneously, leading to a tumor burden of 2 or more per mouse. The *NP* and *N* mice do not show a significant difference in tumor burden (Supplementary Fig. 1b).

Mutant p53 is stabilized in the majority of tumors through multiple mechanisms, driving its gain-of-function (GOF) activities that contribute to more aggressive tumor phenotypes^11,12^. To assess mutant p53 presence in these tumors, we examined p53 stabilization. In *NP* tumors, stabilized p53 was readily detected in cancer-associated fibroblasts. In contrast, p53 staining is largely absent in both the tumor cells and stromal compartments of *N* tumors (Fig. 1b, c).

In addition, the presence of mutant p53 in fibroblasts did not affect the stroma abundance in tumors (Supplementary Fig. 1c, d). While a previous study has shown that *Trp53*-null stroma caused a significant impact on the immune landscape of KrasG12D-driven breast tumors^28^, our model revealed no such effect. The number of infiltrating immune cells—including T cells, natural killer (NK) cells, and macrophages—was low in both *N* and *NP* HER2/Neu+ tumors and showed no significant differences between the groups (Supplementary Fig. 1e).

### The presence of mutant p53 in stromal fibroblasts reprograms gene expression in mammary glands and tumors

To investigate the molecular mechanism of the tumor-promoting role of fibroblastic mutant p53, we next performed RNA sequencing (RNA-seq) on tumors collected from *N* and *NP* mice, designated as T^N^ and T^NP^, respectively (n = 4 per genotype). Additionally, mammary glands were harvested for RNA-seq analysis from tumor-bearing *N* and *NP* mice that did not exhibit malignant lesions (designated as MG^N^ and MG^NP^), as well as from age-matched normal WT mice (MG^WT^). Each group included 3–4 biological replicates.

Differential Expression Analysis revealed distinct gene expression profiles between mammary glands from tumor-bearing mice and those from WT controls (Supplementary Fig. 2). Compared to MG^WT^, 4302 genes were upregulated in MG^N^ and 4929 in MG^NP^, while 3775 and 3469 genes were downregulated, respectively (Supplementary Fig. 2a, b). Venn diagram analysis of these differentially expressed genes identified 499 downregulated and 1426 upregulated genes uniquely in the MG^NP^-MG^WT^ comparison, while 805 downregulated and 799 upregulated genes were found only in the MG^N^-MG^WT^ comparison (Supplementary Fig. 2c, d). Numerous differentially expressed genes identified from both comparisons are linked to cell proliferation pathways such as PI3/AKT/mTOR and TNF-α (Supplementary Fig. 2e), suggesting a tumor-prone or precancerous state in the mammary glands of tumor-bearing mice. This makes these precancerous mammary glands an ideal baseline control for studying the molecular mechanisms underlying HER2+ tumor development in our experimental model.

**Fig. 2:**
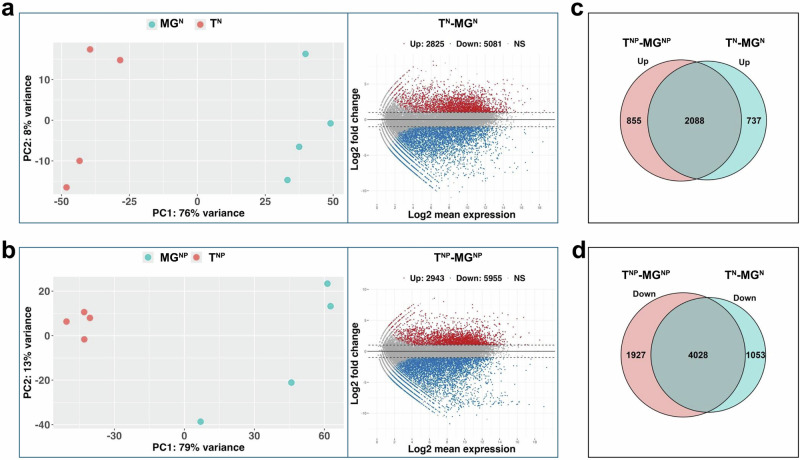
Stromal mutant p53 reprograms gene expression in tumors. **a** Principal component analysis (left) and MA-plot (right) comparing tumors and mammary glands from the *N* mice (T^N^ vs MG^N^). **b** Principal component analysis (left) and MA-plot (right) comparing tumors and mammary glands from the *NP* mice (T^NP^ vs MG^NP^). Red and blue dots in the MA-plots indicate significantly upregulated and downregulated genes, respectively (adjusted *p*-value < 0.05); gray dots represent non-significant changes. **c** Venn diagram depicting the overlap of significantly upregulated genes between T^N^-MG^N^ and T^NP^-MG^NP^ Comparisons. **d** Venn diagram depicting the overlap of significantly downregulated genes between T^N^-MG^N^ and T^NP^-MG^NP^ Comparisons.

To further explore the molecular landscape of the HER2+ tumor progression with WT or mutant p53 in stromal fibroblasts, we next focused on comparing the gene expression profiles between tumors and the matched precancerous mammary glands. Principal component analysis (PCA) showed distinct clustering of tumors and their corresponding mammary gland samples in both *N* (Fig. 2a, left) and *NP* (Fig. 2b, left) mice, implying fundamental transcriptomic differences that may drive the malignant transformation from mammary gland tissue to tumor. To dissect this transformation, we next identified genes that are significantly differentially expressed between tumors and their corresponding mammary gland. 7906 genes were significantly differentially expressed between *N* tumors and *N* mammary gland with a 2-fold change cutoff. Among them, 2825 were upregulated and 5081 downregulated (Fig. 2a, Right, Comparison T^N^-MG^N^). There are 8898 genes significantly differentially expressed in *NP* tumors in contrast to *NP* mammary gland, with 2943 being upregulated and 5955 downregulated (Fig. 2b, Right, Comparison T^NP^-MG^NP^).

To narrow down the genes specifically responsible for fibroblastic mutant p53-mediated non-cell-autonomous effects, we next compared the above two sets of upregulated genes as well as the two sets of downregulated genes in the *NP* and *N* tumors, generated from the T^N^-MG^N^ and T^NP^-MG^NP^ comparisons (Right panels in Fig. 2a, b). 2088 upregulated genes were present in both *NP* and *N* tumors, which likely represent drivers and passengers involved in the MG-to-tumor transformation and tumor growth that are independent of the fibroblastic mutant p53. Therefore, the remaining 855 upregulated genes solely identified in the *NP* tumors theoretically contribute to mutant p53-mediated paracrine effects (Fig. 2c). Likewise, *N* and *NP* tumors shared 4028 downregulated genes. The remaining 1927 downregulated genes, present exclusively in *NP* tumors, are also implicated in mutant p53-mediated paracrine effects (Fig. 2d).

### The presence of mutant p53 in stromal fibroblasts reprograms molecular signaling in tumors

**Fig. 3:**
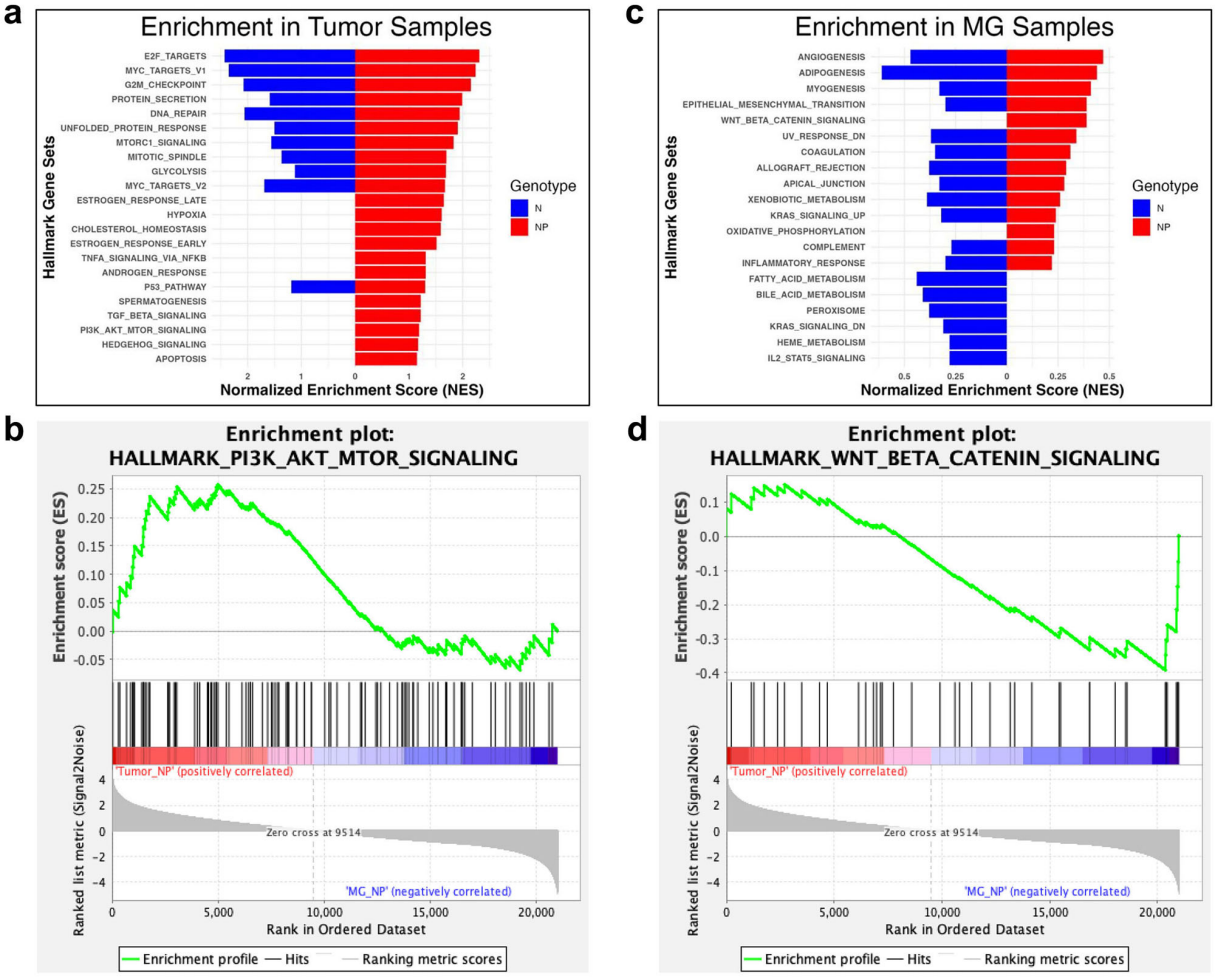
Stromal mutant p53 reprograms molecular signaling in tumors. Gene set enrichment analysis of MSigDB Hallmarks pathways significantly enriched in tumors from *N* and *NP* mice (**a**, **b**) and their corresponding mammary glands (**c**, **d**).

To further understand the biological implications of the genes identified from the T^N^-MG^N^ and T^NP^-MG^NP^ comparisons, we next performed Gene Sets Enrichment Analysis (GSEA) using the mouse Hallmark collection (MH) of the Molecular Signatures Database (MSigDB). The MH collection contains 50 hallmark gene sets with coherent expression patterns, summarizing and representing specific, well-defined biological states or processes^29^. We determined whether any set of genes in this collection shows statistically significant and concordant differences between *NP* tumors and *NP* mammary glands, as well as *N* tumors and *N* mammary glands. All MH gene sets statistically enriched in *N* tumors (FDR q-value < 0.25) overlap with those in *NP* tumors (Fig. 3a). However, *NP* tumors also exhibit additional uniquely enriched sets of genes involved in cholesterol homeostasis, responses of estrogen and androgen, apoptosis, and signaling pathways such as PI3K/AKT/MTOR, TNF-α, TGF-β, and hedgehog (Fig. 3a, b). Additionally, gene sets related to Wnt/beta-catenin signaling and oxidative phosphorylation were specifically enriched in *NP* mammary glands (Fig. 3c, d).

### Fibroblast-expressed mutant p53 upregulates SAA1, SAA2, and THBS4 in *NP* mammary glands, thereby promoting proliferation and migration of ERBB2 tumor cells

**Fig. 4:**
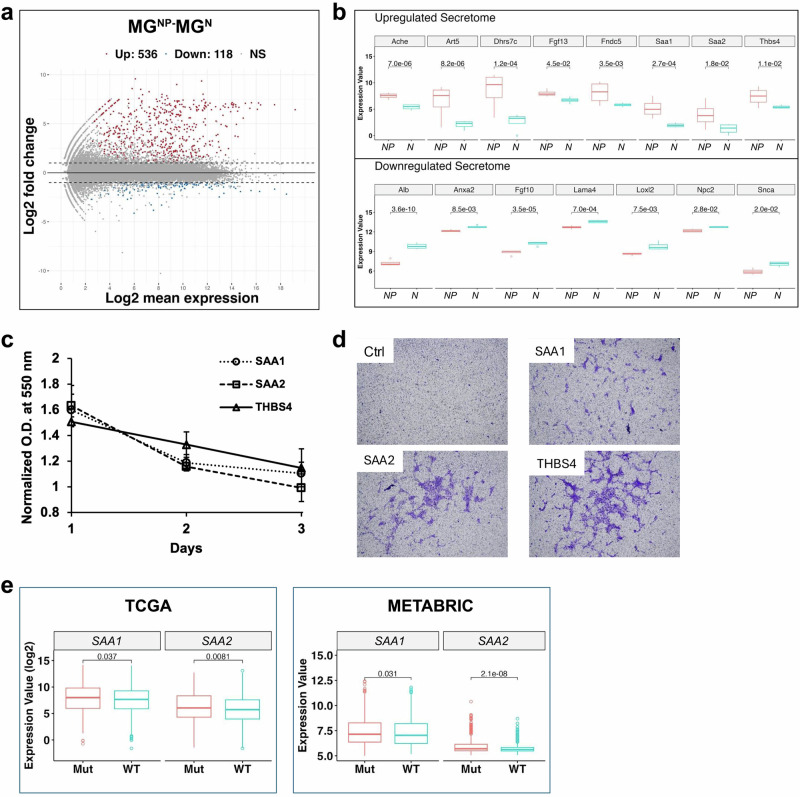
Fibroblastic mutant p53 upregulates SAA1, SAA2, and THBS4 in the *NP* mammary gland, promoting neu tumor cell proliferation and migration. **a** MA-plot comparing mammary glands from *NP* (MG^NP^) and *N* (MG^N^) mice. Red and blue dots indicate significantly upregulated and downregulated genes, respectively (adjusted p-value < 0.05); gray dots represent non-significant changes. **b** Box plots show eight upregulated and seven downregulated secretory genes with adjusted p-values < 0.05. Gene expression levels in MG^NP^ are shown in red, and those in MG^N^ are shown in cyan. **c** MTT assay results showing the effects of secretory peptides on HER2/neu+ mammary tumor cell proliferation. Cells were treated with either vehicle control, SAA1, SAA2, or THBS4 peptides, and cell viability was assessed at 1, 2, or 3 days post-treatment. All comparisons to the vehicle control yielded *p* < 0.05, except for the optical density measured 3 days after SAA2 treatment. **d** Representative images from Boyden chamber assays showing the migration of HER2/neu+ mammary tumor cells in response to secretory peptides, SAA1, SAA2, or THBS4. Ctrl, vehicle control. **e** Transcriptional expression of *SAA1* and *SAA2* in breast cancer samples from the TCGA and METABRIC databases, stratified by *TP53* status (WT, wild-type vs. Mut, mutant).

The above gene sets enriched exclusively in the *NP* tumors (Fig. 3a, b) highlight the signaling events mediated by the paracrine function of fibroblastic mutant p53, which promotes HER2/neu+ mammary tumor development (Fig. 1). To gain a more complete understanding of the paracrine role of mutant p53 in our experimental setting, we next explored which secretory proteins from the *NP* mammary glands may potentially trigger the above signaling in the *NP* tumors. Comparison of the transcriptome of the *N* and *NP* mammary glands generated 654 differentially expressed genes, among which 536 are upregulated, and 118 are downregulated in the *NP* mammary glands (Fig. 4a). Among these 654 genes, fifteen genes possessing secretory features were identified from the Vertebrate Secretome Database VerSeDa^30^. Seven (*Alb*, *Anxa2*, *Fgf10*, *Lama4*, *Loxl12*, *Npc2*, *Snca*) are downregulated in the *NP* mammary gland and eight (*Ache*, *Art5*, *Dhrs7c*, *Fgf13*, *Fndc5*, *Saa1*, *Saa2* and *Thbs4*) are upregulated (Fig. 4b). Among them, in particular, serum amyloid A proteins, SAA1 and SAA2, secreted by cancer cells, cancer-associated fibroblasts, tumor-associated macrophages and adipose cells in the tumor microenvironment, may promote cancer cell proliferation, metastasis and survival. They have been implicated in the pathogenesis of breast, lung, prostate, ovarian, and renal cell cancers^31^. Several other studies have shown that the proliferative effects of these proteins may also be dependent on PI3K signaling^32–35^. In addition, thrombospondin-4 (THBS4) is a non-structural extracellular matrix molecule and has also been shown to promote cancer progression by modulating the PI3K/AKT pathway^36,37^. The remaining secretory genes identified in our analysis have limited evidence supporting their roles in breast cancer development or PI3K signaling based on current literature and were therefore not further pursued. To validate whether these three secretory proteins, SAA1, SAA2, and THBS4, can indeed promote HER2+ tumor development, we next examined the effects of the addition of each of the three synthesized peptides to the HER2+ mammary tumor cell culture derived from our *MMTV-neu* mouse cohorts, thereby confirming that these transcriptomic findings translate into functional drivers of tumor development in this model. Using 3-(4,5-dimethylthiazol-2-yl)-2,5-diphenyltetrazolium bromide (MTT) colorimetric assays, we observed that treatment with SAA1, SAA2, or THBS4 significantly promoted cell proliferation in three HER2+ mammary tumor cell lines derived from our mouse model, with effects evident on days 1, 2, or 3 (Fig. 4c). In the remaining cell line, SAA2 and THBS4 also promoted increased proliferation. The *NP* and *N* tumors exhibited similar metastatic frequency based on microscopic examination under brightfield illumination. However, interestingly, all three peptides, SAA1, SAA2, and THBS4, also enhanced the migration of two out of three HER2/neu+ cells as examined by the Boyden chamber experiments (Fig. 4d), indicating that the presence of mutant p53 in stromal fibroblasts might have endowed higher metastatic potential to the HER2+ mammary tumor. Examining metastasis at an earlier stage could provide insights into metastatic differences in *MMTV-neu* mice, with or without stromal mutant p53.

To determine whether the expression of mutant p53-dependent secretory factors observed in mice is also present in humans, we analyzed data from two human cancer databases: The Cancer Genome Atlas (TCGA) and Molecular Taxonomy of Breast Cancer International Consortium (METABRIC), both of which contain breast cancer transcriptome data. As shown in Fig. 4e, we observed that expression of *SAA1* and *SAA2* is significantly higher in breast cancer samples with mutant *TP53* compared to those with WT *TP53*. Although it remains unclear whether here SAA1 and SAA2 are expressed by stromal or tumor cells—a question best addressed by spatial or single-cell sequencing—these findings further imply the association between mutant p53 and secretory factor expression.

**Fig. 5:**
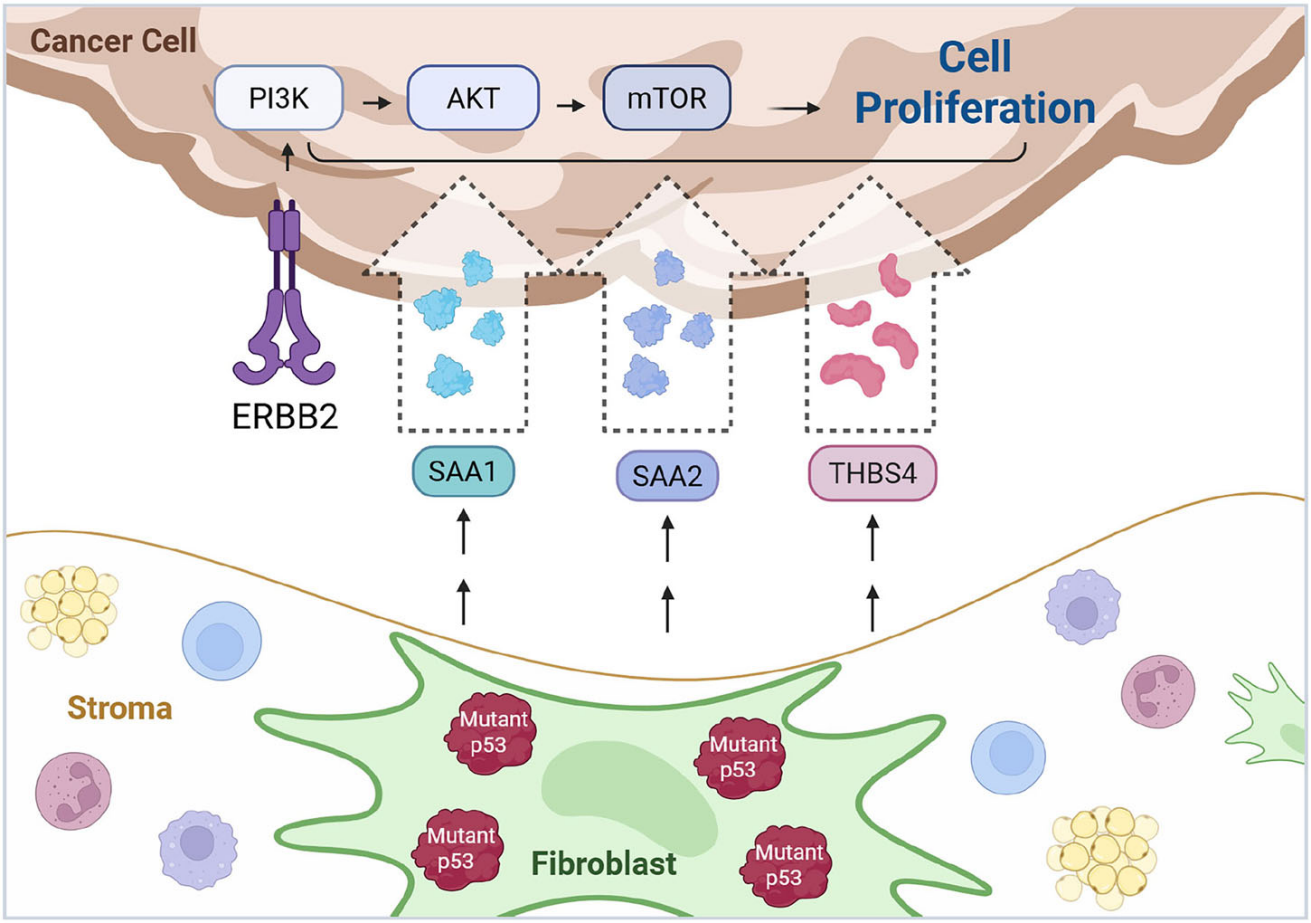
Proposed mechanism by which stromal fibroblastic mutant p53 promotes the development of HER2+ breast cancer. Created with BioRender.com.

Taken together, our data suggests a potential mechanism for the paracrine effect of mutant p53 on breast cancer. Specifically, fibroblastic mutant p53 leads to an increase in secretory factors, including SAA1, SAA2, and THBS4, etc., in the mammary gland. The secretion of these factors, in turn, may activate tumor-promoting pathways such as PI3K signaling in breast tumors, thereby accelerating tumor development (Fig. 5). Although the functional connection between these secreted factors and PI3K pathway activation in our experimental context remains to be validated, our findings suggest that fibroblast-derived signals downstream of mutant p53 may contribute to a tumor microenvironment conducive to cancer development.

## Discussion

Tumor-associated fibroblasts are one of the most dominant and critical components in the tumor stroma. They not only provide physical support for tumor cells but also play diverse functions in matrix deposition and remodeling, extensive reciprocal signaling interactions with tumor cells, and immune modulation. The tumor suppressor *TP53*, the most frequently mutated gene in cancer, was discovered to undergo genetic changes in CAFs. Furthermore, emerging evidence indicates that p53 status in stromal fibroblasts plays an important part in tumor development. Several reports have provided insights into the molecular mechanisms of the non-cell-autonomous role of stromal p53 in cancer development. In two studies, Trp53 was found to attenuate cancer cell migration and invasion through repressing the chemokine SDF-1 and its cognate receptor CXCR4^13,38^ In addition, *TSPAN12*, a p53-regulated gene, was found to be required to enhance cancer invasiveness and proliferation elicited by p53 downregulated fibroblasts^39^. A more recent study highlighted the promoting function of stromal ZEB1/Trp53 signaling axis in mammary epithelial tumor development^40^. Upon ZEB1 deletion in stromal fibroblasts, the acetylation, expression, and recruitment of TP53 to FGF2/7, VEGF, and IL6 promoters are increased, leading to the reduction of their production and secretion into the surrounding stroma^40^. In the present study, we investigated whether and how fibroblastic mutant Trp53 impacts HER2+ mammary tumor development. In contrast to the studies described above, which conducted in vitro assays using fibroblasts of varying *p53* status or involved in vivo deletion of stromal WT *Trp53*, we utilized the conditional *Trp53*^*wm-R172H*^ allele, which allows WT-to-mutant p53 conversion and thus more faithfully mimics the clinical situation of the occurrence of somatic mutant p53. Our study suggests another novel mechanism where mutant p53 in stromal fibroblasts increases the level of secretory proteins such as SAAs (SAA1 and SAA2) and THBS4, triggering tumor-promoting pathways, including PI3K/AKT signaling, etc., in the mammary epithelial cells, which expedites the mammary tumor development. To the best of our knowledge, this is the first in vivo study to propose a mechanism underlying the tumor-promoting function of stromal mutant p53, linking mutant p53-induced secretory factors in stromal fibroblasts to downstream biological pathways in mammary epithelial tumors that drive cell proliferation (Fig. 5).

One remaining question is the precise cellular origin of these secretory proteins. Our RNA-seq analysis revealed that approximately 30% of total *p53* reads in the mammary glands of *NP* mice, which comprise diverse cell types, including fibroblasts, epithelial cells, adipocytes, vascular and immune cells etc., correspond to the *Trp53*^*R172H*^ allele that generates the p53R172H mutant (Supplementary Fig. 3). Assuming equal numbers of fibroblasts and epithelial cells, complete recombination of the *Trp53*^*wm-R172H*^ allele would theoretically yield ~25% *p53*^*R172H*^ reads. Considering that WT p53 from other cell types in the mammary glands, in addition to epithelial cells, would further dilute this signal, the observed ~30% fraction indicates efficient Fsp-Cre–mediated recombination and suggests that fibroblasts constitute a major cell population in the mammary gland. Furthermore, RNA-seq of *N* and *NP* tumors did not reveal differential expressions of *Saa1*, *Saa2*, or *Thbs4*, implying that these proteins are unlikely to originate from epithelial cells in *NP* mice. Nevertheless, it is possible that other stromal populations contribute to their production. Future studies employing single-cell or spatial transcriptomics will be valuable to more precisely dissect the cellular sources of these differentially expressed genes.

It is also noteworthy that in our study, although the presence of fibroblastic mutant p53 expedites neu mammary tumor development, it does not affect tumor metastasis. However, a previous study showed that the presence of either p53-null or p53-mutant fibroblasts evidently increased the rate of metastatic spread of PC3 tumors when PC3 prostate cancer cells were co-inoculated with WT, Trp53-KO, or Trp53-mutant MEFs into the SCID mice^13^. Different cancer types being investigated between these studies may account for the discrepancy. In fact, it was recently shown that stromal p53 regulates breast cancer development in an oncogene-specific manner. Specifically, stromal p53 loss enhances KrasG12D, but not ErbB2, driven tumorigenesis in murine mammary epithelia^28^. In addition, this observation, together with our findings that the fibroblastic mutant p53 promotes ErbB2 tumor development, indicates the gain-of-function of mutant p53 in this scenario. On the other hand, the enhanced migratory response of HER2/neu+ tumor cells to SAA1, SAA2, and THBS4 (Fig. 4c, d) suggests that stromal mutant p53 may predispose tumors to metastasis at earlier stages. Further time-course studies will be needed to elucidate the temporal effects of these factors on metastatic progression. In addition, although *NP* mice exhibited significantly shorter tumor-free survival compared to *N* mice (Fig. 1a), and SAA1, SAA2, and THBS4 enhanced HER2/neu+ cancer cell proliferation in vitro (Fig. 4c), Ki67 staining of tumor sections did not reveal differences in proliferation between *N* and *NP* tumors. One possible explanation is that the tumors analyzed were collected at the endpoint, when proliferation rates may have already plateaued in vivo. This observation also highlights the need for a time-course study in future investigations.

Our analysis of human breast cancer data from TCGA and METABRIC further supports our findings in mice, revealing elevated expression of *SAA1* and *SAA2* in tumors with *TP53* mutations. It remains unclear how mutant p53 increases *Saa1*, *Saa2* and *Thbs4* RNA levels mechanistically, though. Mutant p53 could either directly or indirectly upregulate them, which needs to be experimentally verified with assays including chromatin immunoprecipitation.

In conclusion, our findings underscore the cell non-autonomous role of mutant p53 in cancer, supporting a model in which fibroblastic mutant p53 promotes breast tumorigenesis through paracrine signaling. By inducing the expression of secretory factors that activate oncogenic pathways in neighboring epithelial cells, mutant p53 in the stroma emerges as a key driver of tumor progression. These results highlight stromal mutant p53-mediated tumor–stroma crosstalk as a critical, and potentially targetable, component of breast cancer biology.

## Methods

### Mice

The *MMTV-neu* (stock #002376, FVB background) and *Fsp-Cre* (stock #012641, BALB/c background) transgenic mice were obtained from the Jackson Laboratory. The *p53*^*wm-R172H*^ allele was generated by the Lozano laboratory, previously described^18^, and maintained on a C57BL/6 background. *MMTV-neu* and *Fsp-Cre* mice were crossed with *p53*^*wm-R172H*^ mice to produce *MMTV-neu; p53*^*wm-R172H*^ and *Fsp-Cre; p53*^*wm-R172H*^ mice, which were then used as breeders to generate the experimental cohorts: *MMTV-neu; Fsp-Cre*, *MMTV-neu; p53*^*wm-R172H*^, and *MMTV-neu; Fsp-Cre; p53*^*wm-R172H*^ mice, all maintained on a mixed FVB/C57/BALB/c genetic background. Mice are euthanized using CO₂ inhalation followed by cervical dislocation to collect tumors and tissues. All mouse experiments were performed in compliance with the MD Anderson Cancer Center Institutional Animal Care and Use Committee and conform to the guidelines of the United States Animal Welfare Act and the National Institutes of Health.

### Histology, immunofluorescence, and immunohistochemistry

Tissues were fixed in 10% (vol/vol) formalin and embedded in paraffin by the MD Anderson Research Histology Core Laboratory. Sections were stained with hematoxylin and eosin or trichrome.

Antibodies used for immunofluorescence were p53, 1:300 (Novacastra, NCL-L-p53-CM5p); and alpha smooth muscle Actin 1:400 ([1A4] Abcam 7817). Immunofluorescent secondary IgG antibodies used were Alexa Fluor 555 (Invitrogen A-31572); and Alexa Fluor 488(Abcam 150113); 1:600 for both. DAPI was used for nuclear staining, and slides were mounted with Vectashield HardSet mounting medium (Vector Laboratories H-1400-10). Antibodies used for immunohistochemistry were alpha smooth muscle Actin 1:1000 (Abcam 5694); CD3 1:500 (Abcam 5690); NCR1 1:200 (Abcam 199128); and F4/80 1:200 (Abcam 6640 [CI:A3-1]). Visualization was performed using the ABC and DAB kits (Vector Laboratories), and counterstaining was performed with Mayer’s hematoxylin (Lillie’s modification) (DAKO S3309). Antigen retrieval buffer was either Sodium Citrate pH 6 or Tris-EDTA pH 9.

### RNA sequencing

RNA from tumors and mammary glands was isolated using TRIzol reagent (Life Technologies) and RNeasy Mini Kit (Qiagen). The subsequent RNA sequencing was performed at the Advanced Technology Genomics Core (ATGC) at the M. D. Anderson. Barcoded libraries were prepared using the Illumina TruSeq mRNA seq Kit. The libraries were sequenced on the HiSeq3000 using the 75-bp paired-end format. The raw RNA-sequencing (RNA-seq) readouts were mapped to the mouse assembly reference genome (GRCm38) using TopHat2 and bowtie2^41,42^, an open-source software tool that aligns RNA-seq reads to a reference genome. Differential gene expression analysis was performed with DESeq2 (an R/Bioconductor package)^43^ using adjusted p-value < 0.05 as the significance cutoff. The RNA-seq data are available on the Gene Expression Omnibus (GEO) database with accession number GSE296930. Principal Component Analysis (PCA) was performed using DESeq2 with a regularized log (rlog) transformation of normalized counts.

### Gene set and secretome analyses

The gene set enrichment analysis using the GSEA tool developed by the Broad Institute, which is available at http://www.broadinstitute.org/gsea^44,45^. GSEA utilizes the Hallmark collection of differentially expressed gene sets that summarize and represent specific, well-defined biological states or processes and display coherent expression from the mouse Molecular Signatures Database (MSigDB).

For secretome analysis, the secretory proteins were downloaded from the Vertebrate Secretome Database (VerSeDa). The corresponding Ensembl gene IDs were obtained from Ensembl Biomart.

The normalized gene expression count was generated with the DESeq2 Bioconductor package^46,47^. The heatmap was generated with the differentially expressed genes, and the Pearson distance and the complete linkage clustering were utilized in the analysis.

### Cell proliferation and migration assays

HER2+ tumors were harvested from the *MMTV-neu*; *Fsp-Cre* or *MMTV-neu* female mice when their dimension reached 2 cm. Following isolation with trypsin, primary tumor cells were cultured in Dulbecco’s Modified Eagle Medium (DMEM) containing 10% fetal bovine serum (FBS) and harvested when they reached about 80% confluency, followed by storage in liquid nitrogen.

To measure proliferation, 1 × 10^4^ tumor cells per well were seeded in 96-well plates and cultured in DMEM with 0.05% FBS 24 h before the addition of the peptides. The SAA1 (Aviva Systems Biology, OPCA03381), SAA2 (Aviva Systems Biology, OPCA00215), or THBS4 (Aviva Systems Biology, OPCA07418) peptides were added to the culture medium individually with a final concentration of 100 nM. 48 h following peptide addition, cell proliferation was measured using the 3-(4,5-dimethyl thiazol-2-yl)-2,5-diphenyltetrazolium bromide (MTT) assays as previously described. MTT at 5 µg/mL was incubated in 96-well plates for 3 h, and the optical density was measured at 550 nm.

### TCGA and METABRIC analyses

Breast cancer samples from The Cancer Genome Atlas (TCGA) and the Molecular Taxonomy of Breast Cancer International Consortium (METABRIC) were stratified based on *TP53* status (wild-type vs. mutant). Expression levels of the secretory genes *SAA1*, *SAA2*, and *THBS4* were then statistically compared between groups using the Wilcoxon test.

### Statistical analysis

Mouse survival curves by Kaplan–Meier plots were analyzed by log-rank (Mantel–Cox) tests. Statistical significance was defined as *p* < 0.05.

## Supplementary information

Supplementary Figures and Legends

## Data Availability

The RNA-seq data are available on the Gene Expression Omnibus (GEO) database with accession number (GSE296930).

## Code availability

1. Read Mapping: RNA-seq raw reads were aligned to the reference genome using TopHat (version 2.1.1) with default parameters (https://ccb.jhu.edu/software/tophat/index.shtml). The reference genome (mouse genome assembly GRCm38) and corresponding Ensembl annotation were obtained from the iGenomes repository (https://support.illumina.com/sequencing/sequencing_software/igenome.html). Command used: tophat -p 12 -G ${REF_PATH}/genes.gtf -o ${dir_sample}_thout ${REF_PATH}/genome ${Read1} ${Read2} 2. Read Counting: Gene-level read counts were computed using HTSeq (version 0.11.1) (https://htseq.readthedocs.io/en/latest/index.html). Command used: htseq-count -f bam -s reverse -r name ${bamfile} ${REF_PATH}/genes.gtf > count_output. 3. Differential Expression Analysis: Differentially expressed genes were identified using the DESeq2 R package (version 1.44.0) (https://bioconductor.org/packages/release/bioc/html/DESeq2.html). The detailed analysis workflow is available in the official vignette (https://bioconductor.org/packages/release/bioc/vignettes/DESeq2/inst/doc/DESeq2.html). 4. Functional Enrichment Analysis: Gene Set Enrichment Analysis (GSEA) was conducted using GSEA software version 4.3.2 (https://www.gsea-msigdb.org/gsea/index.jsp). The Mouse Molecular Signatures Database (MSigDB v2025.1) was utilized for gene set definitions. For secretome-specific analysis, a list of secretory proteins was retrieved from the Vertebrate Secretome Database (VerSeDa) (http://genomics.cicbiogune.es/VerSeDa/index.php).
