# Supplementary material for: Stromal fibroblastic mutant Trp53 promotes mammary tumor development via enhanced secretion of paracrine factors
Source: NPJ Breast Cancer. 2025 Dec 12;12:12. doi: 10.1038/s41523-025-00876-y (PMC12800020; doi:10.1038/s41523-025-00876-y)
Supplement: Supplementary file 1 — Supplementary Figures and Legends [file 41523_2025_876_MOESM1_ESM.docx]

**
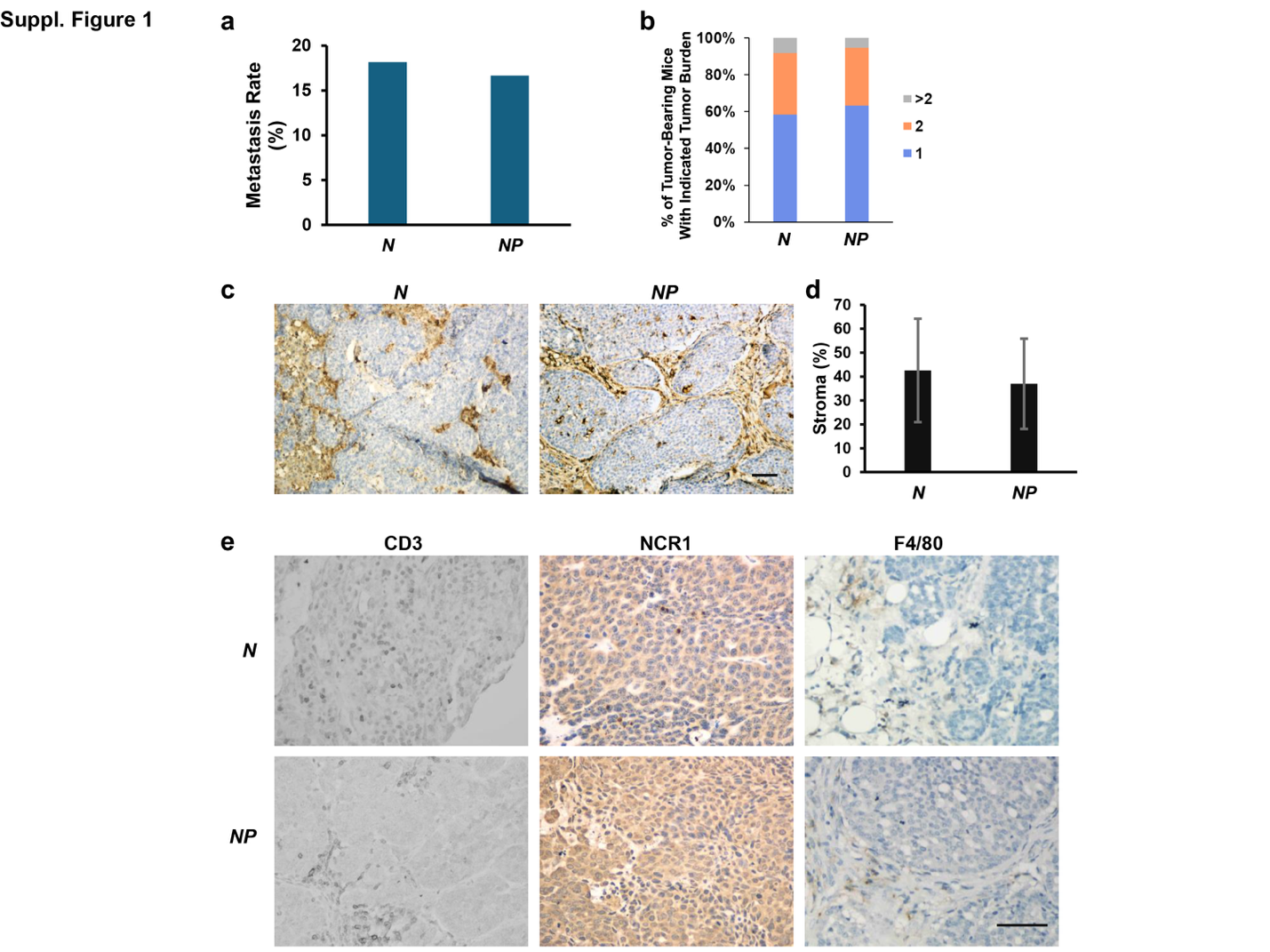
**

**Supplementary Fig. 1: Stroma abundance and immune markers in *N* and *NP* tumors.** **a** Tumor metastasis rate of *N* and *NP* female mice (*p* = 0.93). **b** Tumor burden distribution of *N* and *NP* female mice (*p* = 0.33). **c** Representative immunohistochemistry (IHC) images showing smooth muscle actin (SMA) staining in *N* and *NP* tumors. **d** Quantification of SMA staining. IHC images were randomly captured, and SMA-positive areas were measured using ImageJ and compared between *N* (n = 21) and *NP* (n = 13) tumors (*p* = 0.434). **e** Representative IHC images showing staining for CD3 (T cells), NCR-1 (NK cells), and F4/80 (macrophages) in *N* and *NP* tumors. Scale bar: 50 μm.


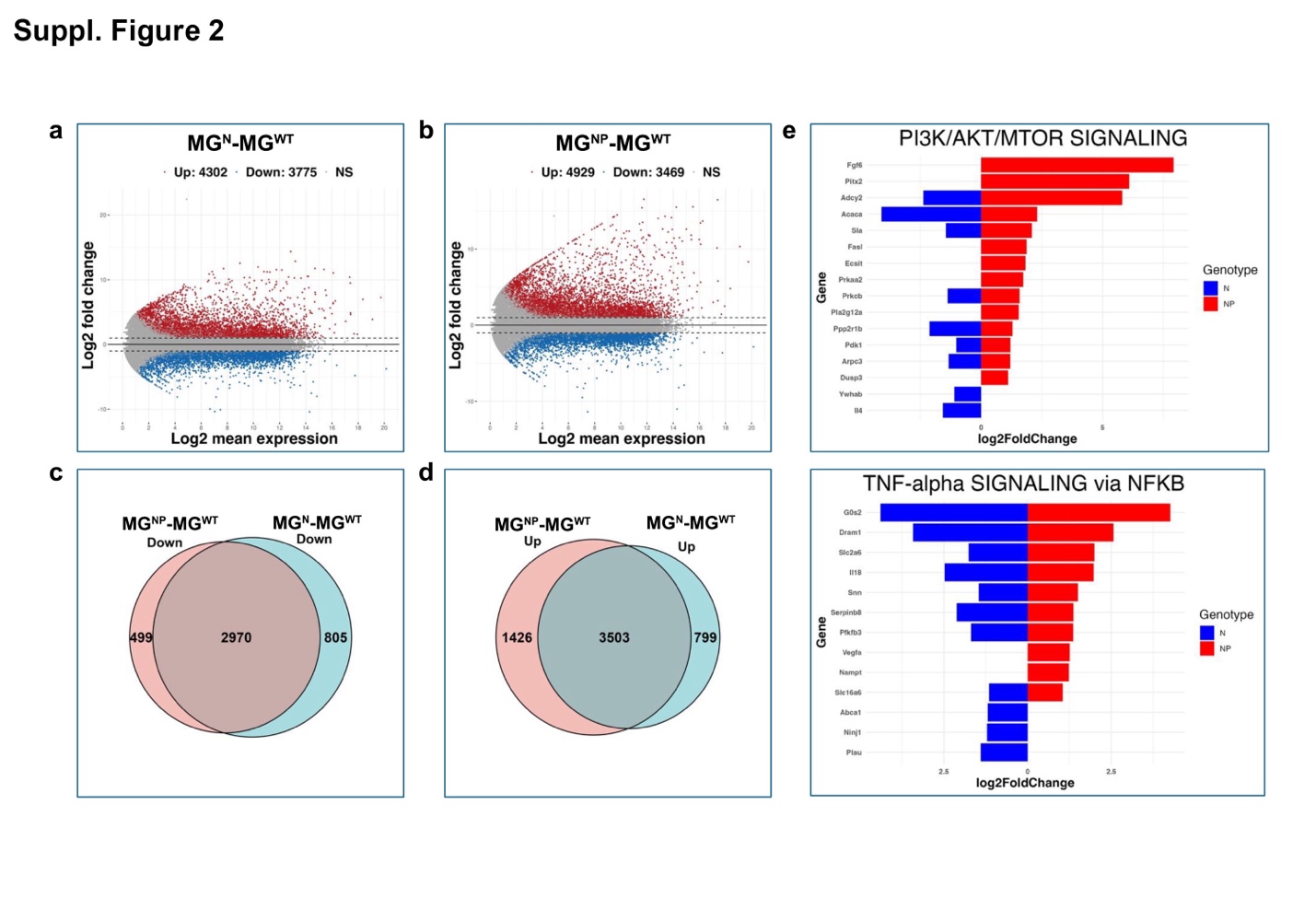


**Supplementary Fig. 2: Transcriptomic comparison of mammary glands from tumor-bearing *N* and *NP* mice with age-matched wild-type mice.** **a** MA plot comparing mammary glands from *N* (MG^N^) and wild-type (MG^WT^) mice. **b** MA plot comparing mammary glands from *NP* (MG^NP^) and wild-type (MG^WT^) mice. Red dots represent significantly upregulated or downregulated genes (adjusted p-value < 0.05), and gray dots represent non-significant changes. **c** Venn diagram illustrating the overlap of significantly downregulated genes between MG^N^ vs. MG^WT^ and MG^NP^ vs. MG^WT^ comparisons. **d** Venn diagram illustrating the overlap of significantly upregulated genes between MG^N^ vs. MG^WT^ and MG^NP^ vs. MG^WT^ comparisons. **e** Genes in PI3K/AKT/MTOR (upper panel) and TNF-alpha (lower panel) signaling pathways that are significantly upregulated in MG^N^ or MG^NP^ compared to MG^WT^.


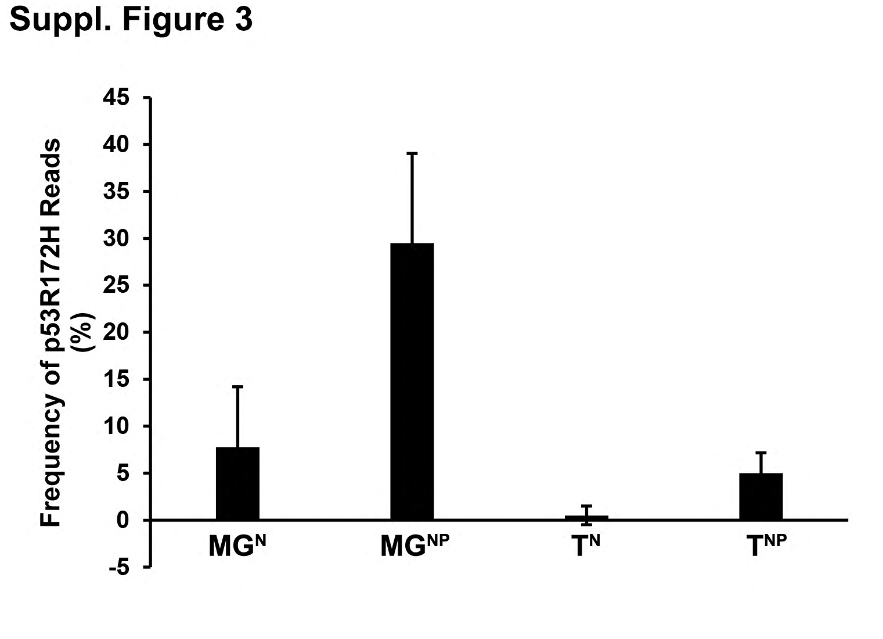


**Supplementary Fig. 3: Frequency of *p53^R172H^* allele reads in mammary glands and tumors.** RNA-seq analysis showing the proportion of *p53^R172H^* reads in mammary glands of *N* and *NP* mice (MG^N^, MG^NP^) and in *N* and *NP* mammary tumors (T^N^, T^NP^). Each bar represents the mean frequency of *p53^R172H^* reads (n=4 per group). *p* < 0.01 for comparisons between MG^NP^ and each of the other three groups.
